# The journey from blue to pink–a rare cause for self-limiting methemoglobinemia in an Indian baby

**DOI:** 10.1515/crpm-2021-0054

**Published:** 2022-08-11

**Authors:** Shanu Chandran, Benjamin J. Ross, Manish Kumar

**Affiliations:** Department of Neonatology, Christian Medical College, Vellore, Tamil Nadu, India

**Keywords:** cyanosis, fetal hemoglobin, methemoglobinemia, newborn

## Abstract

**Objectives:**

To describe a rare case of methemoglobinemia in a newborn baby with excellent prognosis. Methemoglobinemia in the neonatal period is very rare and when present is usually caused by environmental toxicity from strong oxidizing agents and rarely due to enzyme deficiency or inherited disorders of hemoglobin metabolism.

**Case presentation:**

We report a newborn baby presented with cyanosis and desaturation right from birth, later found to have methemoglobinemia and started medication. Genetic evaluation revealed a mutation in the gamma chain of fetal haemoglobin (HbF) causing abnormal hemoglobin. Physiologically significant mutations in gamma-globin genes cause symptoms in the fetus and neonate that gradually abate in the first few months of life.

**Conclusions:**

Genetic evaluation is advisable in babies with unexplained methemoglobinemia as the prognosis of the condition depends on the underlying mutation. Early diagnosis of methemoglobinemia due to gamma chain mutation in HbF as in our case helps in reassuring the parents and also in preventing unnecessary aggressive investigations.

## Introduction

Cyanosis is an ominous physical finding that poses great diagnostic and management challenge, particularly in the newborn period. Though cyanosis commonly occurs in association with neonatal sepsis, cyanotic congenital heart disease, and airway abnormalities, rare and life-threatening causes including methemoglobinemia due to enzyme deficiency, oxidant injury or abnormal hemoglobins should be kept as differential diagnosis [1]. Methemoglobinemia is an uncommon clinical problem in the neonatal period and when present is usually caused by environmental toxicity from strong oxidizing agents and only very rarely from an inherited disorder of hemoglobin metabolism [2]. We came across a cyanotic newborn with the initial diagnosis of methemoglobinemia who was later found to have a mutation in the gamma chain of fetal haemoglobin (HbF) causing abnormal hemoglobin HbF-M. This is the first such case which is reported in an Indian neonate. Physiologically significant mutations in gamma-globin genes (*HBG1 or HBG2*) cause symptoms in the fetus and neonate that gradually abate in the first few months of life.

## Case presentation

A term boy baby with birth weight of 3 kg was delivered by caesarean section in view of fetal distress. He cried immediately after birth. Routine care was given and at 10 min of life, he was noticed to have a low SpO_2_ (oxygen saturation) reading of 75–80%. So, he was started on oxygen by head box at 5 L/min. He had good respiratory efforts with mild tachypnea. As he continued to have low SpO_2_ even after a trial of both 5 L/min of head box oxygen and T piece resuscitator PEEP (positive end expiratory pressure) with 6 cm of water with FiO_2_ (inspired oxygen fraction) 100%, he was intubated and started on positive pressure ventilation. Baby was shifted to the Neonatal Intensive Care Unit for further management.

Even after mechanical ventilation in synchronized intermittent mandatory ventilation (SIMV) mode with maximum stabilizing pressures of PEEP 6 cm and peak pressure 20 cm of water and adequate sedation, the maximum SpO_2_ attained was 80%. He had cyanosis and features of poor perfusion (tachycardia and prolonged capillary refill time). So, the baby was started on inotrope support after a bolus of Normal Saline. Initial differentials considered were septic shock, pneumothorax, lung malformations, and cyanotic heart disease. Blood culture was done and baby was started on first line antibiotics. Chest X-ray showed normal cardiac shadow and normal lung fields. Echocardiogram was done which ruled out persistent pulmonary hypertension and structural heart disease.

Arterial blood gas done after 1 h of ventilation (at 2 h of life) showed pH of 7.5, paCO_2_ 19.1 mmHg, paO_2_ of 199 mmHg, lactate 4 mmol/L, bicarbonate 18.1 mmol/L and base excess of −3.6. As there was unexplained hyperoxemia despite low SpO_2_ and persistent cyanosis, methemoglobinemia was suspected which was confirmed by high metHb (methemoglobin) level (7.6%). Serial blood gas values showed hyperoxemia and high metHb levels (Table 1). He was given 3 mg intravenous single dose methylene blue and started on oral ascorbic acid 300 mg once daily. His ventilator settings were slowly weaned and he was extubated after 16 h to room air. Inotropes were also tapered and stopped within 24 h. As his blood culture had no growth, antibiotics were stopped after 2 days. From day 2 of life, he was maintaining a saturation of 75–85% in room air.

**Table 1:** Trends in metHb and Hb levels.

	**2 h**	3* mg intra-venous single dose Methylene blue*	**12 h**	**Day 2**	**Day 3**	**2 weeks**	**2 months**	**5 months**
MetHb levels	7.6%		5.9%	2.2%	3.7%	3.9%	2.7%	<2%
Hemoglobin	14.5 g%				14.3 g%		10.4 g%	11.5 g%

metHb, methemoglobin; Hb, hemoglobin.

There was no relevant perinatal and family history. Glucose-6-phosphate dehydrogenase level and hemoglobin electrophoresis were also normal. Since day 3, he was on orogastric feeds and was otherwise stable except for persistent cyanosis. After sending genetic test (clinical exome), he was discharged on day 9 while on direct breast feeds as he continued to be stable except for cyanosis and low SpO_2_ reading (80–85%) in room air with normal paO_2_.

After discharge, the baby was under regular follow up and by 2 months of life, his saturation in room air was 88–90% and by 4 months SpO_2_ >92% on room air with no cyanosis. The clinical exome showed mutation in gamma chain of fetal hemoglobin – amino acid substitution of Tyrosine for histidine at codon 63 (p.His63Tyr) due to missense mutation in *HBG2* gene (chr11:g.5275647G>A). Once we received the genetic test result, oral ascorbic acid was stopped and the parents were counselled about the excellent prognosis of this rare disease condition. At 5 months of life, he was well, acyanotic and his metHb level was normal.

## Discussion

Neonatal cyanosis is associated with disorders occurring with deoxygenated hemoglobin, such as heart and airway malformations, hemoglobin variants with low oxygen affinity and methemoglobinemia due to cytochrome b5 reductase deficiency or M-hemoglobin variants [3]. Ours is a case of neonatal cyanosis due to methemoglobinemia caused by a rare hemoglobinopathy affecting gamma chain of HbF.

Methemoglobinemia is due to oxidation of Hb iron moiety from the ferrous to the ferric state, compromising oxygen exchange and oxygen supply to tissues thus causing cyanosis. Under normal circumstances, significant accumulation of metHb is prevented by the action of the enzyme NADH- metHb reductase (also known as cytochrome b5 reductase), keeping metHb levels below 1% [3, 4]. Deficiency of cytochrome b5 reductase is the most common cause of congenital methemoglobinemia.

Hemoglobin production is characterised by two switches embryologically. The first switch occurs at 2 months of gestation when the production of embryonic Hb (Gower 1, 2 and Hb Portland) switches to fetal hemoglobins. HbF, the predominant hemoglobin in the fetus, is a mixture of two molecular species (alpha 2 G-gamma 2, and alpha 2 A-gamma2) that differ only at position 136 reflecting the products of two nonallelic gamma-globin genes. The second switch happens just before birth when HbF switches to major adult HbA (alpha 2 beta2) and minor adult HbA_2_ (alpha 2 delta 2) tetramers. As a result of the second switch, at birth the circulating Hb contains 70–80% of HbF. By six months of age, HbF amounts to less than 5% of the total hemoglobin [5]. By 4–6 months of age, the predominant Hb comprises of alpha and beta chains – HbA, hence the manifestations due to the abnormal gamma chain disappear within this time-frame, as seen in this child.

Neonatal cyanosis associated with Hemoglobin F-M variants is a very rare condition. As per reports in the literature, only six G gamma chain variants were identified (Hb-FM Osaka, Hb-FM Fort Ripley, Hb-FM Circleville, Hb F-Cincinnati, Hb-FM Toms River and Hb Viseu). All of these have single amino acid substitutions in residues crucial to normal heme-globin interaction [6], [7], [8], [9], [10], [11]. The majority of previously discovered HbM and HbF-M variants are caused by the substitution of the distal or proximal histidine residues to tyrosine (Table 2). Although there are 39 differences in amino acid residues between β and γ chains, positions 63 and 92 in both are occupied by a histidine residue and coordinate with heme iron. The imidazole group of normal histidine residues does not form a bond with the heme iron; however, the presence of the tyrosine residue causes the formation of a covalent link between its phenolic moiety and heme iron, so that the heme iron is stabilized in the Fe^3+^ form. When this occurs, oxygen can no longer bind to hemoglobin leading to cyanosis [3]. Our baby had a mutation at site 63 (His63Tyr) in *HBG2* gene which is same as in Hb-FM Osaka which was first detected in a Japanese preterm newborn with cyanosis. This is the first reported mutation in the particular site of gamma chain in an Indian neonate. The family is from Gudiyatham town in the South Indian state of Tamil Nadu. The inheritance of this mutation is autosomal dominant; however, we could not do the genetic studies for the parents as they were not willing.

**Table 2:** Hb F M variants (gamma chain mutations of fetal Hb) associated with neonatal cyanosis.

Codon Change	Nucleotide Position	Amino Acid Change	Hb Variant	References
TTC-TCC	125T>C	Phe41Ser	Hb-F cincinnati	Kohli-kumar et al. [1]
CAT-TAT	190C>T	His63Tyr	Hb-FM osaka	Urabe et al. [7]
CAT-CTT	191A>T	His63Leu	Hb-F circleville	Dainer et al. [3]
GTG-ATG	202G>A	Val67Me	Hb-F toms river	Crowley et al. [8]
CAC-TAC	277C>T	His92Tyr	Hb-FM fort ripley	Priest et al. [2]
CTG-ATG	85C>A	Leu28Met	Hb viseu	Bento et al. [6]
*CAT-TAT*	*190C>T*	*His63Tyr*	*Present case similar to Hb FM Osaka*	

Hb, hemoglobin.

In managing a cyanotic infant, physicians depend on pulse oximetry readings and arterial blood gas values. Some cases of methemoglobinemia can have a normal PaO_2_ and a falsely normal pulse oximetry reading and, in such cases, co-oximeter gives more accurate measurement of oxygen saturation. A co-oximeter measures light absorbance at 4 different wavelengths corresponding to the absorption characteristics of deoxyhemoglobin, oxyhemoglobin, carboxyhemoglobin, and methemoglobin [2].

There is no specific treatment for congenital methemoglobinemia. Administration of oxidative compounds which can cause metHb elevation should be avoided in such cases. In severe methemoglobinemia, methylene blue is used which gets converted to leukomethylene blue in the presence of nicotinamide adenine dinucleotide phosphate (NADPH), resulting in non-enzymatic reduction of MetHb [1]. It is found to have good response in cases of cytochrome b5 reductase enzyme deficiency; but not in HbM. Another drug used is ascorbic acid which is an antioxidant and coenzyme for reduction. If both drugs fail to reduce the MetHb level, hyperbaric oxygen and exchange transfusions are alternative therapies [12]. In our case, we tried methylene blue and ascorbic acid which were later stopped after the genetic diagnosis. We are not sure whether this child had really benefitted from these drugs or not.

## Conclusions

Cyanosis due to high metHb level is rare in the newborn period and the prognosis of the condition depends on the etiology. Those with mutation in the gamma chain of HbF have an excellent prognosis and their symptoms as well as the elevated metHb levels normalize over 4–6 months of life. HbF-M disorders differ from those affecting the alpha chains and beta chains where the metHb level either remains constant or increases over time. Hence, in cases of unexplained methemoglobinemia in newborn period, it is advisable to do genetic testing for definitive diagnosis. Families carrying such gamma chain mutations should be informed in detail about the reason for their babies being cyanosed in early infancy in order to avoid unnecessary aggressive investigations.
